# Determinants of caesarean section use in Ghana: A trend analysis of 2003–2022 DHS data

**DOI:** 10.1371/journal.pgph.0005473

**Published:** 2025-11-14

**Authors:** Johnson Sam, Christian Kasu

**Affiliations:** 1 Department of Population and Health, University of Cape Coast, Cape Coast, Ghana; 2 Essikado Hospital, Sekondi-Takoradi, Ghana; PLOS: Public Library of Science, UNITED STATES OF AMERICA

## Abstract

The global rise in caesarean section (CS) delivery has raised significant public health concerns, particularly regarding drivers and potential health implications. Although the World Health Organization (WHO) recommends CS rates between 5% and 15%, global averages exceed this range, with regional disparities and medically unjustified procedures contributing to the increase. In Ghana, similar trends are emerging amid infrastructural and referral system limitations, yet limited studies have used nationally representative data to track changes over time. This study used data from the 2003, 2008, 2014, and 2022 Ghana Demographic and Health Surveys (GDHS) to examine trends and determinants of CS among women aged 15–49 years who had a live birth in the five years preceding the survey. Weighted descriptive statistics and multilevel logistic regression were employed to estimate adjusted odds ratios (OR) and 95% confidence intervals (CI) for predictors, accounting for the complex survey design. Key variables included survey year, antenatal care (ANC) attendance, maternal education, household wealth, birth size, multiple births, maternal age, and birth order. CS prevalence rose from 4.4% in 2003 to 20.1% in 2022 (p < 0.001). Clinical predictors included multiple births (OR≈5.0), first-order births and larger perceived birth size. Non-clinical factors also strongly influenced CS use: higher education, maternal age ≥ 30 years richest wealth quintile (OR≈3.5), frequent antenatal care attendance, and urban residence increased odds, while rural residence reduced odds. Temporal effects remained strong even after adjustment, underscoring a systemic rise in CS. CS delivery in Ghana has risen sharply over two decades, driven by both clinical and non-clinical factors, including socioeconomic status, education, and healthcare access. Policy interventions should focus on monitoring CS indications, ensuring equitable access, and safeguarding against medically unnecessary procedures.

## Introduction

The rising prevalence of Caesarean Section (CS) deliveries has become a major concern in obstetrics and public health. Globally, CS rates have steadily increased over the past decades, with considerable variations between countries and between public and private hospitals [1,2]. While CS is a surgical procedure involving incisions in the mother’s abdomen and uterus, intended as a life-saving intervention when vaginal delivery poses risks to the mother or baby, the use has expanded beyond medically justified levels. The World Health Organization (WHO) recommends an optimal CS rate of 5–15%, yet approximately 21.1% of births globally now occur via CS [3,4].

Countries such as China (25.9%), Australia (32.3%), and New Zealand (45.9%) report particularly high rates of CS deliveries [2]. Furthermore, prevalence varies by countries in Africa: Uganda (5.2% in 2011), Lesotho (9.7% in 2014), and Nigeria (2.1%) [5]. In Ethiopia, for example, 29.55% of women who gave birth in medical facilities had a CS. The global rise in CS rates has become increasingly evident. A significant contributor to this trend is the growing number of institutional births, along with factors such as the proliferation of unregulated healthcare facilities particularly within the private sector and a rising preference among women for elective CS deliveries [6].

Beyond obstetric necessity, non-clinical factors such as maternal anxiety about vaginal delivery, personal preference, educational attainment, and socioeconomic status influence the likelihood of undergoing CS [7–9]. Medically unjustifiable CS not only adds to healthcare costs but also increases risks of maternal, neonatal, and perinatal complications [2,10]. WHO projections suggest the global CS rate could reach 29% by 2030 [11,12]. The increase has become a major concern in public health due to its associated maternal, neonatal, and perinatal risks, cost, and inequity of access [13,14].

In Ghana, CS prevalence from 2014 Ghana Demographic and Health Survey (GDHS) data was 11.4%, while the Dodowa Health and Demographic Surveillance System reported 6.5% between 2011 and 20135. Between 2015 and 2016, national CS deliveries rose from 14.6% to 16.0%, with increases recorded in all administrative regions except the Upper East Region. The region is one of Ghana’s most rural, dispersed, and infrastructurally challenged regions, but has achieved strong gains in maternal and child health through improved primary care coverage. Ghana’s health system faces infrastructural limitations, with many facilities unable to perform CS and lacking ambulances for timely referrals, leading to poorer outcomes for emergency referrals compared to booked cases [5,7,10].

While previous studies in Ghana have established associations between CS delivery and maternal socioeconomic factors, these analyses were largely based on cross-sectional data from single survey years [5]. Additionally initial analyses employed standard logistic regression to examine individual-level predictors of CS delivery. This study used a multilevel logistic regression model to account for clustering at the community, which introduces contextual depth. The multilevel approach allowed for the estimation of both fixed effects of individual-level variables (e.g., wealth, education, antenatal care visits) and random effects due to unobserved heterogeneity at the cluster level. This is particularly important given the hierarchical structure of DHS data and the potential influence of community- or facility-level factors on maternal health outcomes.

## Materials and methods

### Ethics statement

This study used publicly available DHS data and was exempt from ethical review. Permission to use these datasets was granted by MEASURE DHS following a review of the submitted concept note. The datasets are accessible at: https://dhsprogram.com/data/available-datasets.cfm

### Study design

This study adopted a repeated cross-sectional design using four waves of nationally representative data from the GDHS (2003–2022). The primary focus of the GDHS is on maternal and child health, providing essential data for monitoring health and population trends in Ghana. The survey collects comprehensive information on topics such as fertility, contraceptive use, child health, nutrition, malaria, HIV and AIDS, family planning, health insurance, and maternal healthcare including CS delivery, Antenatal Care (ANC), delivery care, and postnatal care [15].

### Data source

The GDHS was conducted in 2003, 2008, 2014, and 2022 using the standard DHS model questionnaire developed by the MEASURE DHS program [15–18]. The GDHS is a nationally representative survey covering all ten former and sixteen current administrative regions of Ghana. Conducted every five years, the survey is implemented by the Ghana Statistical Service and the Ghana Health Service, with technical assistance from ICF International through the MEASURE DHS program. The GDHS employed a multistage stratified cluster sampling design to ensure national representation [17].

### Study population

For this study, the analysis included women who had a birth history, were currently pregnant, or had given birth within five years prior to the survey and had responded to questions related to ANC attendance. We included women aged 15–49 who had a live birth in the five years preceding each survey. Sample sizes included 5,218 women in 2022, 4,294 in 2014, 2,147 in 2008, and 2,777 in 2003. Women were excluded if they had no live birth within the reference period, if their most recent birth was a stillbirth, or if they had missing data on the outcome variable CS delivery [15–18].

### Variable

The outcome variable of interest was CS delivery; is a surgical procedure used to deliver a baby through incisions in the mother’s abdomen and uterus. It’s typically performed when a vaginal delivery would pose a risk to the health of the mother or baby.

The independent variables in this study were selected based on a review of existing literature on factors associated with CS delivery. These include a range of socio-demographic and obstetric characteristics, grouped into non-clinical and clinical categories for analytical clarity.

Non-clinical (socio-demographic) factors included: number ANC attendance or visits. ANC attendance was defined as having at least one visit; however, it is important to note that the WHO currently recommends a minimum of eight contacts for optimal maternal and neonatal outcomes. Due to data limitations in earlier survey years, ANC attendance in this study was operationalized using the older four-visit threshold, which aligns with Ghana’s current national guidelines. While the WHO now recommends a minimum of eight ANC contacts for optimal maternal and newborn outcomes, this updated standard was not consistently captured across all DHS datasets other variables are place of residence (urban/rural), distance to a health facility (perceived difficulty in accessing care), household wealth index, religion, ethnicity, education level, employment status, marital status and age at first birth. Clinical factors included: multiple births (singleton vs. twins), birth order, and perceived birth size (as reported by the mother) and sex of the child. These variables were included to account for obstetric risk factors that may influence the likelihood of undergoing a CS delivery.

### Statistical analysis

The statistical software STATA version 17 was used to process the data. Some variables were recoded and renamed so that they would be consistent across all the rounds and all results were weighted. Cases with missing values on key variables were excluded from the analysis using listwise deletion. Univariate and multivariate analysis were carried out. Univariate analysis dealt with descriptive statistics, including frequencies and weighted percentages, were used to summarize the background characteristics of the study population. For multivariate analysis, multilevel logistic regression models were employed to estimate the adjusted odds ratios (OR) and 95%, 99% and 99.9% confidence intervals (CI) for the association between CS delivery and its determinates. Two separate models were employed: the first model was for the clinical factors and the second model for the non-clinical predictors. All analyses accounted for the complex survey design using Stata’s “svyset” command.

## Results

### Background characteristics

Table 1 presents the weighted percentage distribution of women aged 15–49 who had a birth in the five years preceding each survey round. The prevalence of CS increased significantly over time, from 4.4% in 2003 to 20.1% in 2022, with an overall pooled prevalence of 13.1%. This sharp upward trend mirrors broader changes in maternal healthcare access and utilisation. In contrast, the proportion of multiple birth remains relatively stable, showing only minor variation over time (from 2.3% in 2003 to 2.6% in 2022 respectively). The proportion of very small perceived birth size reduced over time but fluctuated between 2008 and 2014 (3.9% and 4.7% respectively). Finally, there was no even trend in the sex of the child but overall, the proportion of the male children was higher than female children across the survey years.

**Table 1:** Weighted percentage distributions of sampled women by background characteristics and year of survey.

Characteristic	2003% (n = 2,777)	2008% (n = 2,147)	2014% (n = 4,294)	2022% (n = 5,218)	Pooled % (n = 14,436)
**Cesarean delivery**					
Yes	4.4 (103)	7.2 (138)	13.6 (488)	20.1 (801)	13.1 (1,637)
No	95.6 (2,674)	92.8 (2,009)	86.4 (3,806)	79.9 (4,417)	86.9 (12,799)
**Multiple birth**					
Yes	2.3 (62)	2.5 (53)	2.6 (107)	2.6 (124)	2.5 (362)
No	97.7 (2,715)	97.5 (2,094)	97.4 (4,187)	97.4 (5,094)	97.5 (14,074)
**Birth size**					
Very large	12.0 (319)	22.2 (471)	17.7 (681)	14.7 (678)	16.1 (2,220)
Larger than avg.	29.1 (776)	32.9 (701)	33.4 (1,464)	30.1 (1,470)	31.5 (4,614)
Average	40.6 (1,166)	32.4 (676)	32.8 (1,416)	42.9 (1,914)	37.7 (5,386)
Smaller than avg.	10.7 (306)	8.6 (185)	11.4 (521)	8.2 (418)	9.8 (1,504)
Very small	7.6 (207)	3.9 (94)	4.7 (202)	4.1 (184)	4.9 (712)
**Sex of child**					
Male	50.8 (1,408)	51.8 (1,103)	52.5 (2,234)	51.3 (2,697)	51.6 (7,442)
Female	49.2 (1,369)	48.2 (1,044)	47.5 (2,060)	48.7 (2,521)	48.4 (6,994)
**Birth order**					
1^st^	21.4 (578)	22.3 (457)	22.8 (935)	27.7 (1,226)	24.0 (3,337)
2^nd^	19.3 (506)	20.8 (437)	20.3 (839)	21.9 (970)	20.7 (2,869)
3^rd^	16.3 (436)	16.6 (356)	17.8 (731)	15.9 (765)	16.6 (2,375)
4^th^	12.2 (346)	14.6 (296)	13.9 (606)	12.9 (613)	13.5 (1,945)
**Characteristic**	**2003% (n = 2,777)**	**2008% (n = 2,147)**	**2014% (n = 4,294)**	**2022% (n = 5,218)**	**Pooled % (n = 14,436)**
5th+	31.0 (911)	25.8 (601)	25.2 (1,183)	21.6 (1,090)	25.1 (3,910)
**Antenatal Care Visits**					
No visit	10.0 (325)	5.8 (141)	3.1 (153)	2.1 (109)	4.5 (735)
1–3 visits	20.7 (571)	16.1 (362)	10.0 (446)	9.7 (484)	12.8 (1,916)
4–7 visits	46.6 (1,295)	52.5 (1,137)	55.9 (2,463)	49.4 (2,456)	51.3 (7,633)
8 + visits	22.8 (586)	25.7 (507)	33.3 (1,232)	38.9 (1,615)	31.4 (4,152)
Place of Residence					
Urban	32.7 (817)	40.1 (763)	47.8 (1778)	55.3 (2161)	46.0 (5519)
Rural	67.3 (1960)	59.9 (1384)	52.2 (2516)	44.7 (3057)	54.0 (8917)
**Maternal age at birth**					
<15 yrs	3.0 (87)	3.4 (83)	2.9 (142)	1.9 (86)	2.7 (409)
15–19 yrs	47.8 (1,330)	45.3 (994)	41.7 (1,838)	37.4 (1,818)	42.0 (6,197)
20–24 yrs	38.8 (1,071)	37.6 (791)	36.7 (1,625)	40.9 (1,938)	38.6 (5,643)
25–29 yrs	8.8 (241)	11.4 (221)	14.0 (543)	15.5 (673)	13.1 (1,757)
30 + yrs	1.7 (48)	2.4 (58)	4.7 (146)	4.3 (149)	3.6 (430)
**Maternal education**					
No education	38.7 (1,072)	30.8 (774)	26.0 (1,119)	21.7 (1,528)	27.7 (4,993)
Primary	22.3 (577)	24.4 (508)	19.6 (869)	15.3 (813)	19.4 (2,788)
Secondary	37.9 (897)	42.5 (817)	49.7 (1,839)	53.7 (2,456)	47.7 (6,007)
Higher	1.2 (31)	2.4 (48)	4.6 (169)	9.3 (400)	5.2 (648)
**Religion**					
Christianity	72.5 (1859)	60.5 (1248)	62.6 (2563)	56.0 (2616)	61.9 (8286)
Muslem	17.5 (565)	11.2 (207)	13.9 (522)	14.1 (645)	14.3 (1939)
Traditionalist	3.8 (146)	18.0 (417)	16.8 (885)	24.6 (1682)	17.2 (3130)
Other	6.2 (207)	10.3 (275)	6.7 (324)	5.3 (275)	6.7 (1081)
**Marital status**					
Married	79.4 (2,273)	68.1 (1,513)	61.7 (2,801)	60.7 (3,541)	65.8 (10,146)
Single/Not married	20.6 (504)	31.9 (616)	38.3 (1,493)	39.3 (1,677)	34.2 (4,290)
**Distance to Hospital**					
Far	37.9 (1172)	28.6 (704)	26.5 (1318)	25.0 (1530)	28.5 (4724)
Not far	62.1 (1605)	71.4 (1443)	73.5 (2975)	75.0 (3688)	71.5 (9711)
**Ethnicity**					
Akan	47.1 (1079)	46.5 (836)	47.4 (1643)	38.7 (1432)	44.2 (4990)
Ga/Dangme	7.5 (190)	5.0 (101)	6.4 (198)	6.3 (193)	6.4 (682)
Ewe	12.0 (302)	12.9 (272)	13.2 (476)	10.4 (475)	12.0 (1525)
Guan	2.4 (76)	2.9 (58)	1.9 (104)	3.1 (266)	2.6 (504)
Mole-Dagbani	16.5 (672)	20.2 (549)	17.3 (1551)	23.2 (1654)	19.6 (4026)
Grussi	3.0 (110)	3.0 (116)	3.0 (190)	4.0 (269)	3.3 (685)
**Characteristic**	**2003% (n = 2,777)**	**2008% (n = 2,147)**	**2014% (n = 4,294)**	**2022% (n = 5,218)**	**Pooled % (n = 14,436)**
Gruma	3.5 (123)	4.9 (120)	7.7 (390)	9.8 (677)	7.2 (1310)
Hausa	0.9 (20)	0.9 (17)	1.2 (65)	3.7 (197)	1.9 (299)
Other	7.1 (205)	3.8 (78)	1.9 (77)	0.9 (55)	2.8 (415)
**Maternal Education**					
No form. educ	38.7 (1272)	30.8 (774)	26.0 (1419)	21.7 (1528)	27.7 (4993)
Primary edu.	22.3 (577)	24.4 (508)	19.6 (869)	15.3 (834)	19.4 (2788)
Secondary edu	39.8 (897)	42.5 (817)	49.7 (1837)	53.7 (2456)	47.8 (6007)
Higher	1.2 (31)	2.3 (48)	4.6 (169)	9.3 (400)	5.2 (648)
**Occupational status**					
Working	86.4 (2,422)	86.5 (1,867)	79.4 (3,405)	78.0 (4,011)	81.4 (11,705)
Not working	13.6 (355)	13.5 (280)	20.6 (889)	22.0 (1,207)	16.6 (2,731)

*Source:* Derived from GDHS 2008, 2014 and 2022.

Notes: *p* values from Chi-square tests. *p* < 0.05; *p < 0.01; **p < 0.001. Data weighted to account for sampling design.

Over the study period, several non-clinical factors showed notable changes. There was substantial improvement in ANC attendance, as the proportion of women who report no ANC visit decline sharply, suggesting an enhanced engagement with maternal health service. Urban residency increased steadily from 2003 to 2022, reflecting ongoing urbanisation and possibly improved access to health services in urban areas. Christianity remained the dominate religion across the survey years although its share decline over time. There was also a marked improvement in in perceived access to healthcare, with a growing proportion of women reporting that distance to a health facility was not a barrier. In terms of wealth status, the proportion of women in the poorest wealth quantile decline between 2003 and 2014, with a slight increase in 2022, indicating some fluctuation in poverty levels. Akan was the dominate ethnic group was dominate in all the four survey point, while its proportion declined over time. Changes in maternal age of first birth distribution were also observed: births to girls under 15 decreased, while those to women aged 30 and above increased, highlighting a shift toward later childbearing. Maternal education levels improved markedly. Maternal education levels improved significantly, with sharp decline in proportion of women with no formal education and a notable rise in those attaining higher education. The proportion of married declined substantially, indication a growing prevalence of birth outside of a formal union. Finally, employment remained high but showed a modest decline, suggesting a potential increase in economic inactivity among women who gave birth during the period.

### Multivariate logistic regression of clinical and non-clinical predictors of CS delivery in Ghana

To better understand the distinct contributions of clinical and non-clinical factors to CS delivery in Ghana, we conducted separate multi level logistic regression models for clinical and non-clinical contributor by controlling the survey year. Rather than estimating a single combined model, we fitted two domain-specific multilevel logistic regression models. Model one focused on clinical predictors and the model two focused on non-clinical predictors. This approach was adopted to explore the relative contribution of each domain independently, allowing for clearer interpretation of within-domain effects without the potential for cross-domain confounding. Both models adjusted for survey year to account for temporal trends and included random intercepts at the cluster level to capture unobserved heterogeneity and assess the extent to which the likelihood of CS delivery varied across communities.

### Multi level logistic regression analysis of clinical factors associated with CS delivery in Ghana

Table 2 presents findings from the multilevel logistic regression that included only clinical variables such as multiple birth status, perceived birth size, sex of child, birth order, and survey year as a control variable. The random effect at the cluster level (v001) showed variance of 0.18, indicating a significant clustering effect and suggesting that the likelihood of CS delivery varies across communities. A strong positive association was found between CS delivery and multiple birth (OR ≈ 4.44, p < 0.001), indicating that women with multiple gestations had more than four times the odds of undergoing CS compared to singleton pregnancies. Year of survey was also found to be a strong predictor of CS delivery. Compared with women sampled in the 2003 GDHS, the odds of CS delivery increase in those sampled in 2008 (OR ≈1.56), 2014 (OR ≈2.83) and 2022 (OR ≈4.69) (all p < 0.001), reflecting a clear temporal trend over the study period. Women who perceived their birth size to be smaller than average had 15% lower odds of CS delivery than those with larger than average size but the findings was not significant. Higher birth order was associated with decreasing odds of CS delivery. Compared to the reference category (first birth order), the odds declined from second to third, fourth and fifth, indicating CS delivery is more common in first-time mothers. Giving birth to female infant was associated with 10% lover odds of CS delivery compared to male birth but the difference was not large enough to be significant, and finally, survey year showed increasing odds over time, suggesting temporal trends.

**Table 2:** Multi level logistic regression analysis of clinical factors associated with CS delivery among women in Ghana (GDHS 2003–2022).

*Variable*	*Model OR [95% CI] P-value* *(Random effect (v001): Variance = 0.1342)*
**Multiple birth**	
No (Ref)	1.00
Yes	4.44 [3.42, 5.75] ***
**Birth size**	
Very large (Ref)	1.00
Larger than average	0.63 [0.53, 0.73] ***
Average	0.56 0.48, 0.65] ***
Smaller than average	0.62 [0.50, 0.77] ***
Smaller	0.75 [0.59, 0.99]
**Sex of the child**	
Male (Ref)	
Female	0.90 [0.81, 1.01]
**Birth order**	
1st (Ref)	1.00
2^nd^	0.78 [0.67, 0.91] **
3rd	0.73 [0.62, 0.87] ***
4^th^	0.64 [0.53, 0.77] ***
5^th^+	0.43[0.33, 0.50] ***
***Year of survey***	
2003 (Ref)	1.00
2008	1.56 [1.20, 2.03] ***
2014	2.83 [2.27, 3.56] ***
2022	4.69 [3.77, 5.82] ***

**Notes:** OR = adjusted odds ratio; CI = confidence interval; Ref = reference category. *p < 0.05; **p < 0.01; ***p < 0.001. *Random effect (v001): Variance = 0.18.*

### Multi level logistic regression of non-clinical factors associated with CS delivery in Ghana

As shown in the Table 3 below, A mixed-effects logistic regression model was estimated including fixed effects for ANC attendance, residence, religion, perceived distance to health facility, wealth index, ethnicity, education level age at first birth, marital status, employment status by controlling the year of survey. The random effect at the cluster level (v001) showed variance of 0.18, which means there is a small but significant clustering effect. ANC attendance was significantly associated with higher odds of CS. Women with 4–7 ANC visits had nearly three times higher odds (OR ≈ 2.8, p < 0.001), while those with 8 and above visits had even greater odds (OR ≈2.55, p < 0.001) compared with those who had no ANC visit. Rural residence was associated with significantly lower odds of CS delivery compared to urban residence (OR = 0.86, p < 0.05). Belonging to “other” and “traditional” religious groups (compared to the Christianity) had lower odds of CS delivery (9% and 27% respective) but the difference was not large enough to be significant. Guan ethnicity was associated with reduced odds of CS delivery (OR = 0.72, p < 0.05), as was Gruma ethnicity (OR = 0.60, p < 0.01), relative to the reference ethnic group.

**Table 3:** Multi level logistic analysis of non-clinical factors associated with CS delivery among women in Ghana (GDHS 2003–2022).

*Variable*	*Model OR [95% CI] P-value* *(Random effect (v001): Variance = 0.0306)*
**ANC Attendance**	
No visit	1.00
1-3 visit(s)	1.18 [0.71-1.99]
4-7 visits	2.05 [1.28-3.30] **
8 + visits	2.55 [1.58-4.11] ***
**Place of residence**	
Urban (Ref)	1.00
Rural	0.86 [0.74, 0.99] *
**Religion**	
Christian (Ref)	1.00
Muslem	1.07 [0.91, 1.26]
Traditionalist	0.91 [0.77, 1.09]
Other	0.73 [0.52, 1.02]
**Distance to hospital**	
Not far (Ref)	1.00
Far	1.07 [0.93, 1.22]
**Wealth**	
Poorest (Ref)	1.00
Poorer	1.16 [0.96, 1.41]
Middle	1.42 [1.15, 1.75] **
Richer	1.72 [1.37, 2.16] ***
Richest	2.62 [2.04, 3.37] ***
**Ethnicity**	
Akan (Ref)	1.00
Ga/Dangme	1.15 [0.89, 1.46]
Ewe	1.04 [0.86, 1.25]
Guan	0.72 [0.52, 1.00] *
Mole-Dagbani	0.90 [0.75, 1.07]
Grussi	0.89 [0.66, 1.20] *
Gruma	0.60 [0.45, 1.27] *
Hausa	0.78 [0.52, 1.19]
Other	0.83 [0.54, 1.27]
**Age at Birth**	
Less than 15yrs (Ref)	1.00
15-19 yrs.	0.90 [0.60, 1.34]
20-24 yrs.	1.09 [0.73, 1.63]
25-29 yrs.	1.46 [0.97, 2.21]
30 and above	3.35 [2.13, 5.26] ***
**Maternal Education**	
No formal education (Ref)	1.00
Primary education	1.25 [1.03, 1.52] *
Secondary education	1.46 [1.23, 1.73] ***
Higher	1.99 [1.54, 2.56] ***
**Marital Status**	
Married (Ref)	1.00
Single/not married	0.91 [0.80, 1.03]
**Occupational Status**	
Working (Ref)	1.00
Not working	0.92 [0.83, 1.08]
***Year of survey***	
2003 (Ref)	1.00
2008	1.66 [1.27, 2.18] ***
2014	2.87 [2.28, 3.61] ***
2022	5.04 [4.01, 6.33] ***

**Notes:** OR = adjusted odds ratio; CI = confidence interval; Ref = reference category. *p < 0.05; **p < 0.01; ***p < 0.001. *Random effect (v001): Variance = 0.03.*

A clear socioeconomic gradient was observed with wealth: individuals in higher wealth categories had progressively higher odds of CS delivery. For instance, those in the richest group had nearly three times the odds compared to the poorest (OR = 2.62, p < 0.001,). Similarly, maternal education was positively associated with CS delivery. Compared to those with no education, mothers with higher education had nearly double the odds (OR = 1.99, p < 0.001). Age at first birth also showed a strong effect: women whose first birth occurred at age 30 or above had significantly higher odds of Cs delivery compared to those who gave birth before age 15 (OR = 3.35, p < 0.001). Finally, the odds of CS delivery increased across survey years, with 2022 showing the highest increase (OR = 5.56, p < 0.001), indicating a temporal trend in the likelihood of the outcome.

### Comparative Insight

The clinical model explained more between-cluster variance (0.18) than the non-clinical model (0.03), suggesting that community or facility-level factors more strongly shape the application of clinical criteria for CS delivery. In contrast, non-clinical predictors showed strong individual-level associations, particularly socioeconomic status and education, but less clustering, indicating these factors are more uniformly distributed in their effect across communities.

## Discussions

Comparing the two domain-specific models, the clinical model explained more variance at the cluster level (variance = 0.18) than the non-clinical model (variance = 0.03), suggesting that clinical factors are more influenced by community-level or health system-level variations. The magnitude of association was also stronger for some clinical predictors such as multiple gestations and perceived birth size. However, non-clinical predictors like ANC attendance, maternal education, wealth, and age at first birth were also significantly associated with CS delivery, demonstrating that socioeconomic and demographic factors are key contributors. Together, these findings suggest that both clinical needs and social positioning shape the use of CS in Ghana, albeit in different ways.

The findings from the multilevel logistic regression analysis revealed a multifaceted picture that align with broader regional and global trends. The nearly fivefold increase in CS rate from 2003 to 2022 mirrors global trends, but its trajectory in Ghana appears particularly steep. This trajectory suggests a systemic shift in obstetric norms, potentially driven by institutional incentives, defensive medicine, or investments that prioritize surgical readiness over risk reduction. While CS remains a life-saving intervention, its rapid expansion raises concerns about medicalization of childbirth [3–5]. Although CS as a mode of delivery is an essential surgical process to reduce the risks associated with childbirth, an increasing trend in CS delivery has continued to boost worries worldwide [19,20]. Strong association between multiple births (twins) and CS aligns with established clinical guidelines that prioritize CS for high-risk pregnancies to reduce perinatal morbidity and mortality [12]. The inverse relationship between birth order and CS use suggests that first-time mothers are more likely to deliver by CS, potentially due to longer labour durations, lack of previous delivery experience, or clinical caution during the first delivery [5]. Interestingly, the size of the baby at birth also emerged as a significant factor. Babies categorized as average or smaller than average were less likely to be delivered by CS compared to very large babies. This finding suggests that larger feutal size is a critical consideration in clinical decision-making, consistent with earlier studies that identify macrosomia as a key risk factor for obstructed labour and CS delivery [10].

For the non-clinical factors, the analysis identified maternal age as a significant predictor of CS delivery, with older women, particularly those aged 30 years and above, being more likely to undergo the procedure. This finding is corroborated by earlier studies which indicate that advanced maternal age is associated with increased obstetric risk and a higher likelihood of medical indications for CS [9,12]. Similarly, higher education was strongly associated with CS delivery. This could represent a proxy health literacy and empowered decision making, but it may also reflect differential treatment by providers, with educated women being taken more seriously or viewed as more likely to request for CS [8,19]. These findings raise questions about equity in provider-patient interaction and the potential for socioeconomic status to shape not only access to care but also the trajectory of medical decision-making within the health system.

Wealth status also showed a strong and positive association with CS delivery; women is the richest wealth quantile had nearly threefold higher odds of undergoing CS than those in the poorest group. This gradient is reflective of findings across low- and middle-income countries where socioeconomic disparities influence access to surgical obstetric care. In private healthcare settings, where CS may be financially incentivized, wealthier women may have greater access or may opt for CS even in the absence of medical indications [1,2]. Alternatively, demand-side dynamics such as fear of labor pain, scheduling convenience, or perceptions of CS as superior care may be driving patient preferences in affluent groups. This supports the argument that inequities in healthcare access and provider practices contribute to unjustified variations in CS use [10,13].

These socioeconomic and demographic factors highlight the role of non-clinical determinants, which the WHO has recognized as increasingly influential in the global rise of CS rates [6]. While clinical factors such as multiple gestation and birth order remain strong and legitimate drivers of CS, the influence of social determinants calls for caution. These findings underscore that CS use in Ghana is not purely a reflection of medical need, but is shaped by the interplay between individual status, health system dynamics, and community context. The analysis further found that women who 4–7 and 8 and above ANC visits had the higher odds of CS delivery. This could be possibly due to ANC as a proxy for greater engagement with the formal health system, which in turn increases the likelihood of a medically discretionary CS. This may reflect the dual role of ANC in both increasing access to health services and facilitating early identification of complications can be avert with CS [7]. It was evident in other studies that number of ANC visits during pregnancy has significantly influenced the mode of delivery, where the CS rate was the highest for the mothers having more ANC visits [19]. However, it may also reflect increased exposure to institutional norms and medical advice. These results underscore the complex interplay between clinical needs and social determinants in shaping CS use in Ghana. While CS remains a vital, life-saving intervention, the marked influence of non-clinical factors raises concerns about potential overuse and inequitable access. This aligns with WHO’s warning on the medicalization of childbirth and the growing role of provider and patient preferences in driving CS rates [13,14].

## Conclusions

This study provides updated on the determinates of CS delivery in Ghana using domain-specific multilevel models to disentangle the role of clinical need and social positioning. While the rising CS trend partly reflect medical necessity, particular among high-risk, it also a signal of growing inequalities and potential overuse among wealthier, more educated, and more health-system engaged women. Future interventions should focus on equitable availability, strict clinical justification, and public health strategies that prevent unnecessary surgical deliveries without compromising maternal or neonatal outcomes.

### Policy and clinical relevance

These findings point to the need for a balanced approach that safeguards access to CS for women in need while discouraging unnecessary procedures. Policy actions should include:

1. Implement equity-focused audits of CS deliveries disaggregated by socioeconomic status, facility type (public/private), and indication.
2. Strengthen clinical decision-making protocols, ensuring CS is used based on medical necessity rather than social preference or provider convenience.
3. Training healthcare providers on respectful maternity care and bias mitigation to avoid privileging certain patient profiles in surgical decisions.
4. Integrate ANC services with shared decision-making tools to educate mothers on both the benefits and risks of CS, countering normalisation of unnecessary interventions.
5. Monitor private sector CS trends separately, given the potential financial incentives that may influence provider behavior.

### Limitations

This study has several limitations. First, it used secondary, cross-sectional DHS data, which limits causal interpretation. Second, several variables such as perceived birth size and distance to health facilities were self-reported, which may introduce recall or reporting bias. Third, facility-level and provider-specific factors that could influence CS decisions were not available. Finally, although survey weights were applied to improve representativeness, underreporting or misclassification of delivery mode cannot be ruled out.
